# Immunomodulatory Mechanisms and Therapeutic Potential of Mesenchymal Stem Cells

**DOI:** 10.1007/s12015-023-10539-9

**Published:** 2023-04-14

**Authors:** Guoqiang Yang, Xuehui Fan, Yingchun Liu, Pingping Jie, Maryam Mazhar, Yong Liu, Nathupakorn Dechsupa, Li Wang

**Affiliations:** 1 Research Center for Integrated Chinese and Western Medicine, The Affiliated Traditional Chinese Medicine Hospital of Southwest Medical University, Luzhou, China; 2 Molecular Imaging and Therapy Research Unit, Department of Radiologic Technology, Faculty of Associated Medical Sciences, Chiang Mai University, Chiang Mai, Thailand; 3 Acupuncture and Rehabilitation Department, The Affiliated Traditional Chinese Medicine Hospital of Southwest Medical University, Luzhou, China; 4 Key Laboratory of Medical Electrophysiology, Ministry of Education and Medical Electrophysiological Key Laboratory of Sichuan Province, Collaborative Innovation Center for Prevention of Cardiovascular Diseases, Institute of Cardiovascular Research, Southwest Medical University, Luzhou, China; 5 First Department of Medicine, Medical Faculty Mannheim, University Medical Centre Mannheim (UMM), University of Heidelberg, Mannheim, Germany; 6 Department of Magnetic Resonance Imaging, the Affiliated Traditional Chinese Medicine Hospital of Southwest Medical University, Luzhou, China; 7 National Traditional Chinese Medicine Clinical Research Base and Drug Research Center of the Affiliated Traditional Chinese Medicine Hospital of Southwest Medical University, Luzhou, China; 8 Institute of Integrated Chinese and Western Medicine, Southwest Medical University, Luzhou, China

**Keywords:** Mesenchymal stem cells, Immunomodulation, Therapeutics, Engineered, Immune cells

## Abstract

Mesenchymal stem cells (MSCs) are regarded as highly promising cells for allogeneic cell therapy, owing to their multipotent nature and ability to display potent and varied functions in different diseases. The functions of MSCs, including native immunomodulation, high self-renewal characteristic, and secretory and trophic properties, can be employed to improve the immune-modulatory functions in diseases. MSCs impact most immune cells by directly contacting and/or secreting positive microenvironmental factors to influence them. Previous studies have reported that the immunomodulatory role of MSCs is basically dependent on their secretion ability from MSCs. This review discusses the immunomodulatory capabilities of MSCs and the promising strategies to successfully improve the potential utilization of MSCs in clinical research.

**Graphical Abstract:**

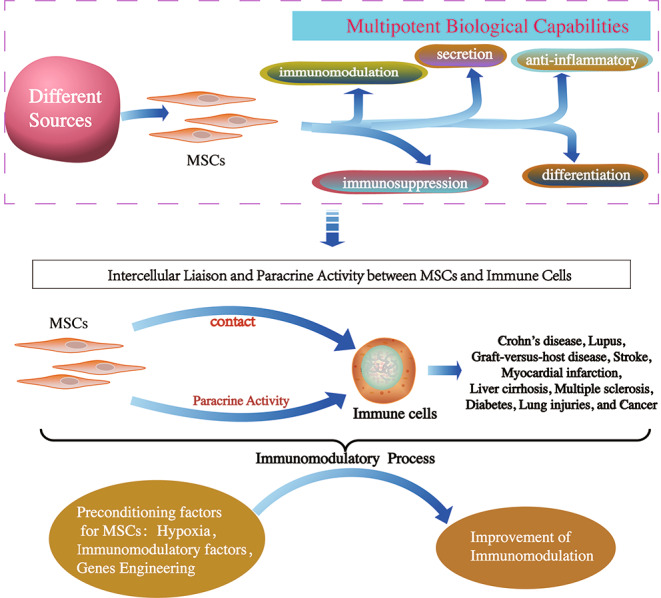

## Introduction

Mesenchymal stem cells (MSCs) were first defined in the late 1960s, as fibroblast-like cells derived from bone marrow (BM) possessing multipotential for high differentiation and self-renewal [1–4]. Later, Caplan et al. found that BM-derived fibroblast-like cells also possess osteogenic and chondrogenic abilities, and named these cells “MSCs” in 1991 [5, 6]. Then, MSCs have been found to differentiate into several other mesenchymal tissues, such as myocytes and adipocytes [7, 8]. Further investigations revealed that MSCs could differentiate into different lineages, including epithelial, neuronal, astrocytic morphology, endothelial, and smooth muscle in vitro, depending on specific conditions of cocultured cell type [9–14]. Meanwhile, Dazzi et al. and Taechangam et al. suggested that the multipotent capacity, support of hematopoiesis in the BM, and their immunoregulatory effects positively endow MSCs treatment as a promising therapeutical strategy [15, 16].

Many studies have reported that MSCs could be isolated from adipose tissue [14, 17]. It has been shown that adipose MSCs (AMSCs) with multipotentiality give rise to adipocytes, neurocytes, chondrocytes, myocytes, and osteoblasts cell lineages [18, 19]. Other reports found that exosomes derived from AMSCs can alleviate the inflammation response to promote wound healing and suppress osteoarthritic cartilage degeneration [20, 21]. MSCs restore mucosal immunity and rejuvenate mucosal immunosenescence in the elderly [22]. Moreover, reprogramming AMSCs into islet β-cells (reprogrammed AMSCs-derived islet β-cells) with low immunogenicity achieved ideal therapeutic effects in treating canine diabetes mellitus [23].

MSCs also can be isolated from neonatal tissue, including human umbilical cord blood (HUCB-MSCs), umbilical cord MSCs (HUC-MSCs), amniotic fluid (HAF-MSCs), placenta MSCs (HP-MSCs), and amniotic membrane (HAM-MSCs), which have been utilized in treating neurological deficits in animal models and patients with intracerebral hemorrhage (ICH) [24–30]. Recently, a report from a clinical trial found that it is safe and well-tolerated to infuse HUC-MSCs intravenously for moderate and severe coronavirus disease 2019 (COVID-19) patients, which may be regarded as a promising therapy in targeting the underlying aberrant immune responses [31]. The transplantation of HUC-MSCs with immunomodulatory, and anti-inflammatory properties emerge in immune and inflammatory diseases and is known as a perspective in future therapeutic utilization [32].

It has been shown that MSCs can be isolated from synovial fluid (SF-MSCs) [33], dental pulp (DMSCs) [14, 34], peripheral blood [25, 35], lungs [36–38], muscle [39, 40] and other sources. Previous studies suggested that the administration of SF-MSCs as a viable therapy is utilized in cartilage degeneration of osteoarthritis (OA) [33, 41, 42]. DMSCs improve the immunoregulatory effects on T and B lymphocyte responses based on decreasing CD4^+^ T lymphocyte proliferation, intracellular Interferon-gamma (IFN-γ), and IL-17 secretion in primary Sjögren’s syndrome [43]. MSCs display different functions for different organs, differentiating into other cell lineages to improve inflammatory response and tissue damage [44–48]. Therefore, MSCs are significant mediators in sustaining tissue homeostasis and improving tissue integrity.

MSCs derived from adult tissues, such as bone marrow, peripheral blood, adipose tissue, etc.) and prenatal tissues (particular parts of the umbilical cord and placenta) have some differences in their cell biological properties, proliferative capacities and surface marker expression. The prenatal tissues with various advantages, including the availability, avoided invasive procedures and ethical problems are widely utilized in the preclinical and clincal research. Baksh et al. found that the HUC-MSCs have a higher proliferation capacity compared with the BMSCs [49]. Moreover, more reports suggested that HUC-MSCs show a higher proliferation capacity than BMSCs [50, 51]. It has been reported that HP-MSCs exhibit a better engraftment and expansion and capacity compared with BMSCs [52]. In recent years, MSCs from other sources, such as AMSCs, HUCB-MSCs, HUC-MSCs, and HPMSCs, have received considerable attention due to better immunomodulatory characteristics and proliferation rate as compared to BMSCs [53, 54]. MSCs can be identified by distinguishing a specific panel of positive cell surface antigens ( CD29, CD73, CD90, and CD105) and negative cell surface markers (CD14, CD11b, CD19, CD34, CD45, CD79α, and HLA-DR). Moreover, multiple differentiation potentials to the osteoblasts, adipocytes, and chondroblasts is a central standard for defining MSCs in vitro [55–57]. MSCs display crucial function in the repairation of damaged tissue by mechanisms of immunosuppression, anti-apoptotic, anti-inflammatory, anti-fibrotic, pro-angiogenic, antitumorigenic, neuroprotective, antibacterial, and chemo-attractive effects [58–60]. These unique characteristics endow MSCs as a promising candidate in regenerative medicine [61], and with absorbing therapeutic potential in inflammatory diseases [58, 62], and cancer [63, 64].

Initially, MSCs were primarily utilized as a promising cell therapy for tissue repair in regenerative medicine [59, 65]. It has been shown that they have been approved in clinical research for autoimmune diseases, including Crohn’s disease, lupus, and graft-versus-host disease (GVHD) [58, 66]. Likewise, MSCs have been investigated for therapeutic potential against different diseases, including stroke, myocardial infarction, liver cirrhosis, multiple sclerosis, diabetes, lung injuries, and cancer, both in preclinical and human translational studies [58, 59]. Moreover, MSCs as potential treatments possess immunomodulatory functions except for being multi-potent, which have been investigated for various immune diseases. MSCs robustly exert their immunomodulation through direct cell–cell contact and their secretion ability with immune cells in the innate and adaptive immune systems [67]. Up to now, the immunomodulatory function of MSCs across tissue sources and species has been well-reproduced and clinically relevant [68]. In this review, we summarise the recent immunomodulatory properties of MSCs in different diseases and discuss the potential therapeutic targets in various diseases.

## Immunomodulation of MSCs

Recent findings indicated that MSCs conduct immunomodulatory activity, associated with monocytes and regulatory T cells (Tregs) through the special cytokine-independent mechanism [58, 69–71]. AMSCs display more potential immunomodulatory effects than BMSCs, suggesting that AMSCs could serve as a better alternative for immunomodulatory therapy [54, 69]. In comparison, HUC-MSCs have shown a minimal response to the allogeneic immune risk in vivo, regarding HUC-MSCs as a suitable therapeutic candidate [72]. Some reports also found that xenogenic human MSCs have the potential to display positive ability in mouse tumor diseases, sheep tibia bone defect, porcine osteochondral reconstitution, and cartilage regeneration in canines [73–75]. Xenogenic human MSCs with lower weakly immunogenicity possess the therapeutic potential in preclinical and clincal studies. Therefore, we summarize the immunomodulatory effects of naive and modified MSCs and review the current understanding of their immunomodulatory aspects.

### Immunomodulation via Intercellular Liaison

**Fig. 1:**
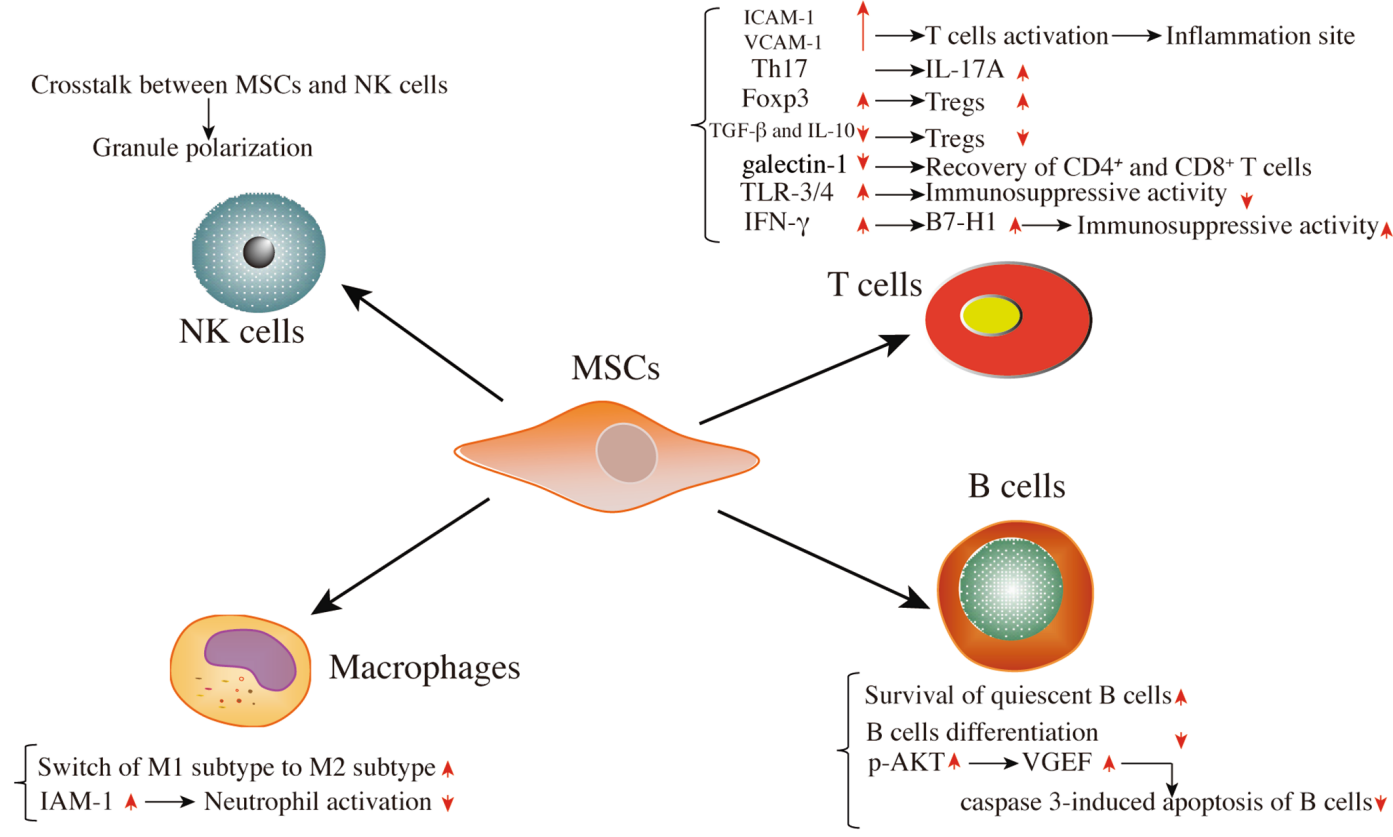
**Cell-to-cell contact of immunomodulation in adaptive immunity and innate immunity**. MSCs display the immunomodulatory effects mainly via mutual effect with immune cells such as T cells, B cells, natural killer (NK) cells, macrophages, etc. and cell-to-cell contact is associated with the modulation of protein expression. Abbreviations: Foxp3, Forkhead box P3; ICAM-1, intercellular adhesion molecule-1; VCAM-1, vascular cell adhesion molecule-1; TLR, Toll-like receptors; IFN, interferon; B7-H1, an inhibitory surface molecule in stem cells; VEGF, vascular endothelial growth factor; IAM-1, intercellular adhesion molecule-1; p-AKT, phosphorylated protein kinase B; TGF-β, transforming growth factor-β; IL, interleukin; B7-H1, an inhibitory surface molecule in stem cells

Many preclinical studies have reported that the administration of MSCs is involved in the innate and adaptive immune responses through communication with the immune cells comprised of T cells, B cells, neutrophils monocytes, macrophages, natural killer (NK) cells, and dendritic cells (DCs), exerting immunomodulatory effects (Fig. 1) through intercellular liaison and paracrine action [76–78].

### Intercellular Liaison in Adaptive Immunity

As shown in in-vitro experiments, BMSCs physically hindered naive and memory-T cell reactions from associating with antigen-presenting cells (APCs) in a noncognate fashion [79]. This function was exhibited through the secretory enhancement of vascular cell adhesion molecule-1 (VCAM-1) and intercellular adhesion molecule-1 (ICAM-1), in favor of T cells activation and leukocytes recruitment to the inflammation site, which plays a critical role in immunosuppressive capacity [80, 81]. Furthermore, BMSCs co-cultured with activated T cells induce Th17 lymphocytes expressing interleukin-17 A (IL-17 A) [82]. The activation of the Notch1/forkhead box P3 (FOXP3) pathway was observed in CD4^+^ T cells co-cultured with MSCs, mediating regulatory T cells (Tregs) induction by increasing the amount of CD4^+^ CD25(high) FOXP3^+^ cells [83, 84]. However, inhibition of TGF-β and IL-10 simultaneously suppressed Tregs induction in co-cultured cells, indicating that the two aspects display an essential effect in the immune-tolerance mechanism [84]. Furthermore, galectin-1 protein is primarily expressed in MSCs, influencing cytokine secretion in GVHD and autoimmunity. The immunomodulatory properties of MSCs to the allogeneic T cells were lost after the knockdown of galectin-1, contributing to the recovery of CD4^+^ and CD8^+^ T cells [85, 86]. Also, the high Toll-like receptors (TLRs), including TLR-3 and TLR-4, are expressed on the membrane of MSCs, the function of whose ligands is responsible for the activity of nuclear factor kappaB (NF-κB) and the secretion of pro-inflammatory cytokines, including CXCL10, IL-6, and IL-8. Expression of TLR-3 and TLR-4 in MSCs effectively inhibited the immunosuppressive activity of MSCs, resulting in efficient T cells response in dangerous infections, such as RNA viruses and bacteria infections [87].

It has been shown that the function of T cells is critical to the immunomodulation of MSCs in vivo mice models. MSCs derived from compact bone (CB) have synergistic anti-tumor efficacy in combination with an immune-activating fusion protein which is associated with the activation of CD4^+^ and CD8^+^ T cells and the inhibition of Tregs in the microenvironment of a syngeneic orthotopic ovarian cancer mouse model [88, 89]. In the lipopolysaccharide (LPS) and immune response-mediated abortion models, MSCs through cell-to-cell contact with the proinflammatory macrophages boost the inhibitive adjusting and proliferation of T cells and switch macrophages to M2 phenotype, mediating abortion relief, demonstrating MSCs potential application in treating the recurrent miscarriages in clinical research [90, 91]. IFN-γ is derived from activated T cells and is regarded as a renowned proinflammatory cytokine. The IFN-γ on MSCs induces the enhancement of an inhibitory surface molecule named B7-H1 on MSCs, enhancing the MSCs’ immunosuppressive properties. On the contrary, primed MSCs with activated T cells from IFN-γ ^−/−^ mice showed a remarkably weakened capacity to restrain the proliferation of T cells in MSCs’ immunosuppressive function through the mechanism of intercellular liaison [92].

Besides T cells, there is an important connection between MSCs and B cells through intercellular liaison. Human-derived AMSCs are based on contact-dependent mechanisms to improve the survival of quiescent B cells and suppress B cells differentiation dependent on T cells [93, 94]. Based on improved phosphorylated protein kinase B (p-AKT) by MSCs within the B cells, AMSCs enhance the secretion of vascular endothelial growth factor (VEGF) to inhibit the caspase 3-induced apoptosis of B cells [95]. Besides, MSCs can block the proliferation of B cells by inhibiting the cell G0/G1 phase cycle of B cells, associated with the phosphorylated extracellular response kinase (p-ERK) and p38 pathways [96].

### Intercellular Liaison in Innate Immunity

Except for the adaptive immune system, MSCs also act on the innate immune system based on the mechanism of intercellular liaison. Tracking studies indicated that viable HUC-MSCs appeared in the lungs directly after intravenous injection. Most HUC-MSCs were dead and then quickly phagocytosed by monocytes after 24 h. Cocultured studies of MSCs either using a transwell or directly with two kinds of NK cell lines demonstrated that granule polarization is either induced or restrained through the special crosstalk of MSCs on the two NK cell lines [97]. Moreover, some studies found that AMSCs can switch an activated-M1 pro-inflammatory phenotype of macrophages to its anti-inflammatory M2 phenotype associated with the specific function of prostaglandin E2 (PGE2) [98, 99]. Furthermore, MSCs hold substantial therapeutic promise to prevent unrestrained neutrophil activation-induced tissue damage depending on the neutrophils’ engulfment of intercellular adhesion molecule-1 (IAM-1) derived from MSCs [100, 101].

Various known mechanisms through which MSCs’ exert immunomodulatory activity are illustrated in Fig. 1. However, it require extensive preclinical studies to be translated in clinical research.

### Immunomodulation via Paracrine Activity

#### The Secretomes of MSCs

MSCs also exert or convey regulatory messages displaying their immunomodulatory functions by releasing secretomes [76, 78, 102]. This secretome derived from MSCs possessing immunomodulatory functions consists of a battery of cytokines, growth factors, chemokines, and extracellular vesicles (MSCs-EVs), which interact with immune cells of the innate and adaptive systems and regulate the immune and cancer-cell function [67, 103–106]. According to their size and origin, encapsulated paracrine molecules in MSCs-EVs are usually divided into exosomes (30-120 nm) from the multivesicular endosomes, microvesicles (MVs) (100–1000 nm) originating from the plasma membrane, and apoptotic bodies [67, 102, 107]. Interactions between MSC-EVs and immune cells can be regarded as an ideal therapeutic prospect for inflammatory, infectious, and autoimmune diseases [108]. Although MSC-EVs are similar to the parent MSCs in the immunoregulatory functions [108], the paracrine function of the MSCs is based on the appropriate cultured conditions [109]. Therefore, it is critical to know the paracrine function of MSCs to improve their immunomodulatory effects (Table 1).

**Table 1:** The secretomes of MSCs

cytokines	chemokines	Growth factors	Other factors
IFN-γ, TGF-β, TNF-α, LIF, MIF, OSM, G-CSF, M-CSF, GM-CSF FLT3LG, SCF, Thrombopoietin, TSG-6, IL-1α, IL-1β, IL-2, IL-3, IL-6~8, IL-10-13, IL-16,	CCL1, CCL2, CCL5, CCL8, CCL11, CCL15, CCL16, CCL18, CCL22~24, CCL26, CXCL1-3, CXCL5, CXCL6, CXCL8, CXCL11~13, CX3CL1, XCL1	BDGF, NGF, GDNF, PIGF, PDGF, VEGF, HGF, EGF, IGF-1, FGF-2, FGF-4, FGF-7, FGF-9, BMP-7	phospholipids, Adrenomedullin, Adiponectin, Osteoprotegerin, miRNAs, mRNAs, long non-coding RNAs, CXCR3, PGE2, PAI-1, MMP1, MMP3, MMP9, MMP10, MMP13, TIMP-1~4, Leptin, IGFBP-1~4

### The Paracrine Function of MSCs in the Adaptive Immune System

MSCs exert immunomodulatory properties on the adaptive immune system depending on their paracrine secretion. MSCs restrain the differentiation of T helper 17 (Th17) cells by mediating the secretion of IL-10, PGE2, and trimethylation of histone to regulate inflammation, which was enhanced in coculturing with Th17 cells and inhibiting expression of TNF-α, IL-17, IL-22, and IFN-γ derived from Th17 cells [110, 111]. It has been shown that both BMSCs priming with cytokines and cell ratio (BMSCs/T cells), moderated their cytokine profiles on the generation of Th17 lymphocytes [82]. Nevertheless, the mechanisms of the mutual effect of MSCs with Th17 cells are not incompletely resolved. Wang et al. have found that modified MSCs with IL-25 knockdown did not mediate Th17 suppression in vitro and in vivo experiments, further verifying that the regulation of the IL-25/STAT3/PD-L1 axis can be identified as a candidate therapeutic target [112, 113]. Indoleamine 2,3-dioxygenase (IDO), secreted by MSCs, is known to suppress T cells and mediate Tregs generation, partly in charge of inducing tolerance of kidney allograft [114]. Moreover, PD-1 ligands (PD-L1 and PD-L2) derived from MSCs restrain the secretion of IL-2 and activation of CD4^+^ T cells and mediate irreversible hyporesponsiveness and cell death to exert immunosuppressive potential on induction of peripheral tolerance and T cell behavior for the treatment of immunological disorders [115].

### The Paracrine Function of MSCs in the Innate Immune System

MSCs communicate with NK cells through the acute suppression of IL-2-mediated resting NK cell proliferation and improve NK function of the innate immune system [116, 117]. Moreover, MSCs block the cytotoxic activity or cytokine production associated with the key mediators, including IDO and PGE2 [118, 119]. It has also been shown that MSCs improve the capacity of IL-12/IL-18-motivated NK cells secreting IFN-γ that can potentially contribute to defending infections for tissue reconstruction at the injured spot [120]. Additionally, MSC-derived IL-6 protects neutrophils from apoptosis, signals via the activation of STAT-3 to preserve their effector functions, and prevents oxidative metabolism in the BM niche [121]. MSC-derived exosomes (MSCs-EX) mainly improve neutrophil viability, whereas MSCs-conditioned media (MSCs-CM) essentially improve the neutrophil’s function, suggesting that both MSCs-EX and MSCs-CM are beneficial for increasing immunity through the improvement of neutrophil’s function and survival [122]. MSCs derived from human BM and salivary gland stimulated by LPS boost the anti-microbial functions of neutrophils by releasing high levels of macrophage migration inhibitory factor (MIF) and inflammatory cytokines (IL-6 and IL-8) and eliminating the infection and inflammation [123].

MSCs show more potent immunomodulatory effects due to having higher levels of multiple cytokines secretion, including IL-6 and TGF-β1, to suppress the proliferation of stimulated PBMCs, and the differentiation of immature DCs derived from monocyte and induce the IL-10 secretion by monocytes [124, 125]. However, MSCs were empowered by MSCs-derived PGE2 to restrain the differentiation of monocytes to mature myeloid DCs, and NS-398, the PGE2 inhibitor, inhibited PGE2 production and restored DC differentiation and function [126]. It has been shown that MSCs-EVs prevent DC maturation and inhibit antigen uptake by immature DCs resulting in the downregulated expression and secretion of mature and activated DCs markers (CD38, CD80, and CD83), upregulated production of anti-inflammatory cytokine TGF-β, and pro-inflammatory cytokines (IL-6 and IL-12p70) [78]. MSCs-EVs, known as effective therapeutic agents, display a crucial function in eliciting macrophage to anti-inflammatory M2 polarization possessing effective anti-inflammatory properties by decreasing expression levels of IL-23 and IL-22 [127, 128]. Activated BMSCs with TNF-α or LPS reprogram macrophages via the higher release of PGE2, affecting the macrophages associated with the receptors of PGEP2 and PGEP4, whereas, both PGEP2 and PGEP4 receptor antagonists inhibited the increased secretion of IL-10 [129]. Human PMSCs can transit macrophages into a typical anti-inflammatory M2 phenotype from a pro-inflammatory M1, which is associated with the secretion changes of IL-1β, IL-10, IL-12p70, and macrophage inflammatory protein-1 alpha (MIP-1α) and partly induced by soluble molecules, including progesterone and glucocorticoid receptors [130].

**Fig. 2:**
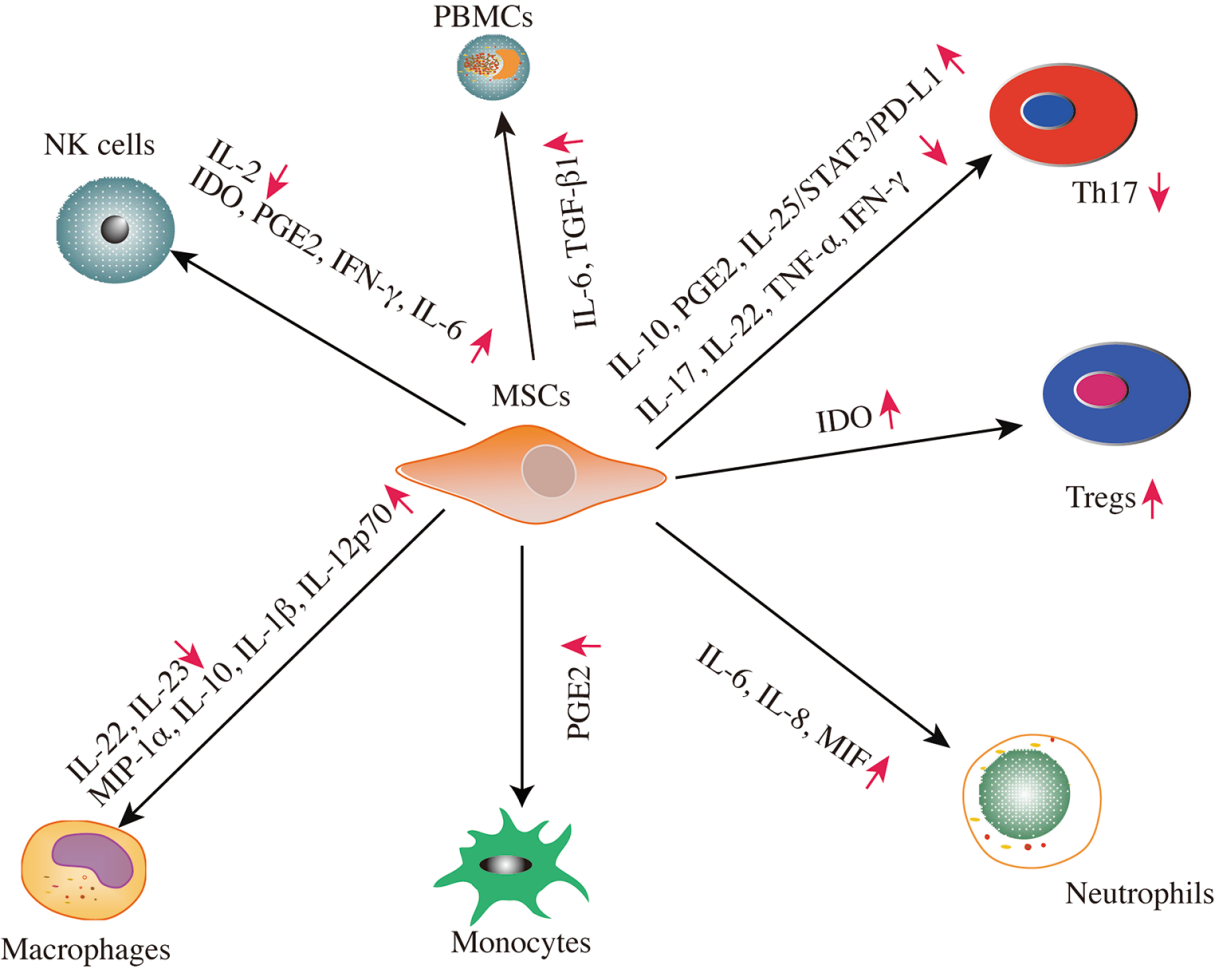
**Paracrine activity of immunomodulation in adaptive immunity and innate immunity**. MSCs exert immunomodulatory effects through the interactions with immune cells such as T cells, natural killer (NK) cells, macrophages, monocytes, PBMCs and neutrophils, through paracrine activity. MSCs’ secretome secret a battery of cytokines, growth factors, and chemokines for playing their immunomodulatory function. Abbreviations: PBMCs, peripheral blood mononuclear cells; IL, interleukin; TGF-β1, transforming growth factor-β1; PGE2, prostaglandin E2; IFN, interferon; MIF, macrophage migration inhibitory factor; IDO, Indoleamine 2,3-dioxygenase; Th17, T helper 17; Tregs, regulatory T cells; MIP-1α, macrophage inflammatory protein-1 alpha

Different secretomes derived from MSCs comprised of cytokines, growth factors, and chemokines have participated in adaptive and innate immune systems to exert immunomodulatory effects (Fig. 2).

## Improvement of Immunomodulatory Capabilities and Therapeutic Efficacy by Modified MSCs

Increasing evidence has demonstrated that preconditioned MSCs can improve their paracrine potency and therapeutic abilities. It has been shown that MSCs pretreated with hypoxia, growth factors, heat shock protein (Hsp), LPS, pharmacological agents, chemical agents, serum deprivation, or inflammatory stimulation, have the ability to improve their potency and survival by increasing the expression levels of cytoprotective genes, and secretion of reparative factors [131, 132]. As such, preconditioning conditions can modulate MSCs’ secretomes in vitro culture [102]. It is critical to analyze the influence of secretome on varied biological processes, including immunomodulation, angiogenesis, anti-fibrotic, neurogenesis, wound healing, tissue repair, and anti-tumor for tissue repairation and regeneration [107].

### MSCs Preconditioning with Hypoxia

Preconditioned MSCs treatment with hypoxia has shown to have therapeutic potential on the MSCs’ immune phenotype. Specifically, MSCs preconditioned with hypoxia increase paracrine effects, especially the production of angiogenic factors and HGF observed in pulmonary fibrosis and acute kidney injury [133, 134]. Overexpressing hypoxia-inducible factor 1 α (HIF-1 α) of dental MSCs (HIF-MSCs) can inhibit DCs differentiation, resist NK-cell-induced lysis to facilitate the therapeutic ability of HIF-MSCs, and improve more monocytes migration to differentiate into suppressor macrophages [135]. Hypoxia-preconditioned CB MSCs (HP-CB-MSCs) possess effective potency to restrain T cell proliferation, including total (CD3^+^), CD4^+^, and CD8^+^ T cells. HP-CB-MSCs, accompanied by preconditioning of selective proinflammatory cytokines, such as IL-17 A TNF-α, and IFN-γ, increase superior inhibition of CD8^+^ T cells compared with CD4^+^ T cells [136]. Under hypoxia conditions, MSCs improve the production of SDF-1α and VEGF, and alter the secretion of angiogenic signals (IL-8, uPA, Angiogenin, and Serpin-1) for the response of hypoxia, which contributes to more endothelial cells infiltrating into an MSCs bio-artificial scaffold for dermal regeneration [137]. Moreover, MSCs from human anticoagulated whole BM are positively impacted by hypoxic culturing on the transcriptome and cell fitness, possibly boosting inherent properties for developing cellular therapies in treating the orthopedic diseases [138]. MSCs derived from rat BM facilitate the characteristics of stem cells accompanied by upregulation of IDO and promote the expansion of Tregs for immunosuppressive function when MSCs are cocultured with CD4^+^/allogeneic endothelial cells [139]. Additionally, primed MSCs by hypoxia and calcium ions (HC-MSCs) have demonstrated a resistance to passage-dependent senescence, which is induced through the p53/p21 cascade and monocyte chemoattractant protein-1 (MCP-1) and improved a large secretion of pro-angiogenic and immunomodulatory factors, contributing to inhibiting the proliferation of T cell. Transplantation of HC-MSCs effectively moderated allogeneic conflicts in the utilization of a humanized GVHD mice model by improving survival and weight loss and reducing histopathologic damage in target organs of GVHD [140, 141].

### MSCs Preconditioned with Immunomodulatory Factors

Another positive approach for pretreatment is priming with immunomodulatory factors. Modified MSCs with TNF-α and IFN-γ can totally reverse the immunomodulatory potency associated with the MSCs’ pro-inflammatory effect in palmitate in vitro [142]. TNF-α and IFN-γ, the key cytokines involved in MSC activation, could improve the expression of immunoregulatory molecules for communication through cell-cell contact and EVs [143]. Three-dimensional spheroidal MSCs primed with the heparin-microparticle transfer of IFN-γ induce continuing expression of IDO, enhancing the immunomodulatory capability, which suppresses the activation and proliferation of T cells [144]. MSCs primed with IFN-γ have enhanced immunosuppressive capacity and immunosuppressive function, suppressing the proliferation of T cells through the IFN-γ/JAK/STAT1 pathway [145, 146]. Moreover, membrane particles (MPs) derived from pretreated MSCs by IFN-γ (MP-MSCs-γ) can enhance the percentage of CD90^+^ and anti-inflammatory PD-L1 monocytes, and the mRNA expression of PD-L1 in monocytes. The results demonstrated that MSCs-derived MPs possess the immunomodulatory capability and potential and can be regarded as a novel therapeutic strategy in treating immunological disorders [147]. Recently, Bolhassani et al. have demonstrated that BMSCs transfected with small Hsp 27 (sHsp27) and E7 oncoprotein DNA vaccinations (E7ODV) restrain tumor growth and increase the responses of E7-specific T cells in a tumor mouse model. This study suggested that MSCs-based vaccinations with particular modifications have the potential to treat HPV-associated cancers and can be an effective strategy for immunotherapy and protective function [148].

### Engineered MSCs Improve the Immunomodulation

Many studies have reported that MSCs possessing intrinsic capacities can be used in immune-associated diseases. As for utilizing MSCs for targeting diseases, investigators have focused mainly on gene-delivery vehicles, like IFNs and ILs, and oncolytic viruses (OV) to improve the immunomodulatory function of MSCs, empowering them to target biologics.

It has been shown that engineered MSCs with the IFN-β improve the survival of glioblastoma (GBM) mice, resistant CNS malignancies, via enhancing CD8 T cells’ selective postsurgical infiltration and directly inducing tumor cells’ cell-cycle arrest [149, 150]. It has demonstrated that it is essential to develop cancer immunotherapies syngeneic for tumor resection models and highlight the translational potential of local delivery of cellular immunotherapy for cancer treatment [149]. Similarly, Relation et al. also found that engineered MSCs with pro-inflammatory cytokine IFN-γ not only deliver IFN-γ directly to the tumor microenvironment but also avoid systemic toxicity, which decreases tumor growth rate and increases survival via inflammatory M1 macrophage polarization (the increased IL-17 and IL-23p19, M1 polarization markers) in vivo and in vitro [151]. Likewise, spheroidal MSCs transfected with a potent anti-inflammatory cytokine IL-4 enhanced anti-inflammatory effects and chondroprotective in OA rats model and in an OA chondrocyte model [152], whereas MSCs engineered anti-tumor activity cytokine, IL-12, prolong the tumor-bearing mouse survival and decrease tumor growth after intravenous injection, including carcinoma, renal cell carcinoma (RCC), breast tumor, and melanoma [73, 153]. Systemic administration of MSCs engineered with lentivirus vector expressing murine IL-15 significantly improved the retardation of syngeneic mouse pancreatic tumor and melanoma growth and prolonged the survival in tumor-bearing mice. Additionally, such cured mice resisted the pancreatic tumor rechallenge [154, 155]. Likewise, genetically engineered MSCs with IL-21 promoted the induction of NK cells and effector T cells and potently restrained the immune suppressor cells, which prevented the tumor nodules in a mouse model of disseminated B cells lymphoma [156]. Additionally, it has been shown that in the streptozotocin-induced diabetic rats model, the injection of stromal cell-derived factor-1 (SDF-1)-modified MSCs (SDF-1-MSCs) can moderate erectile dysfunction. The beneficial effects of SDF-1-MSCs were associated with the enhancement of protein expression levels, including p-AKT, AKT, phosphorylated neuronal nitric oxide synthase (p-nNOS), nNOS, VEGF, B-cell lymphoma-2 (Bcl-2), basic fibroblast growth factor (bFGF), and decline of protein expression levels of apoptosis factors, including the caspase-3 and Bcl-2-associated x (Bax) [157]. Besides, exosomes of AMSCs infected by miR-199a lentivirus have successfully delivered miR-199a to hepatocellular carcinoma (HCC) cells, and remarkably improved sensitivity of chemotherapeutic agents to cancer cells for the improvement of HCC chemosensitivity via the inhibition of the mammalian target of rapamycin (mTOR) signaling pathway [158].

**Fig. 3:**
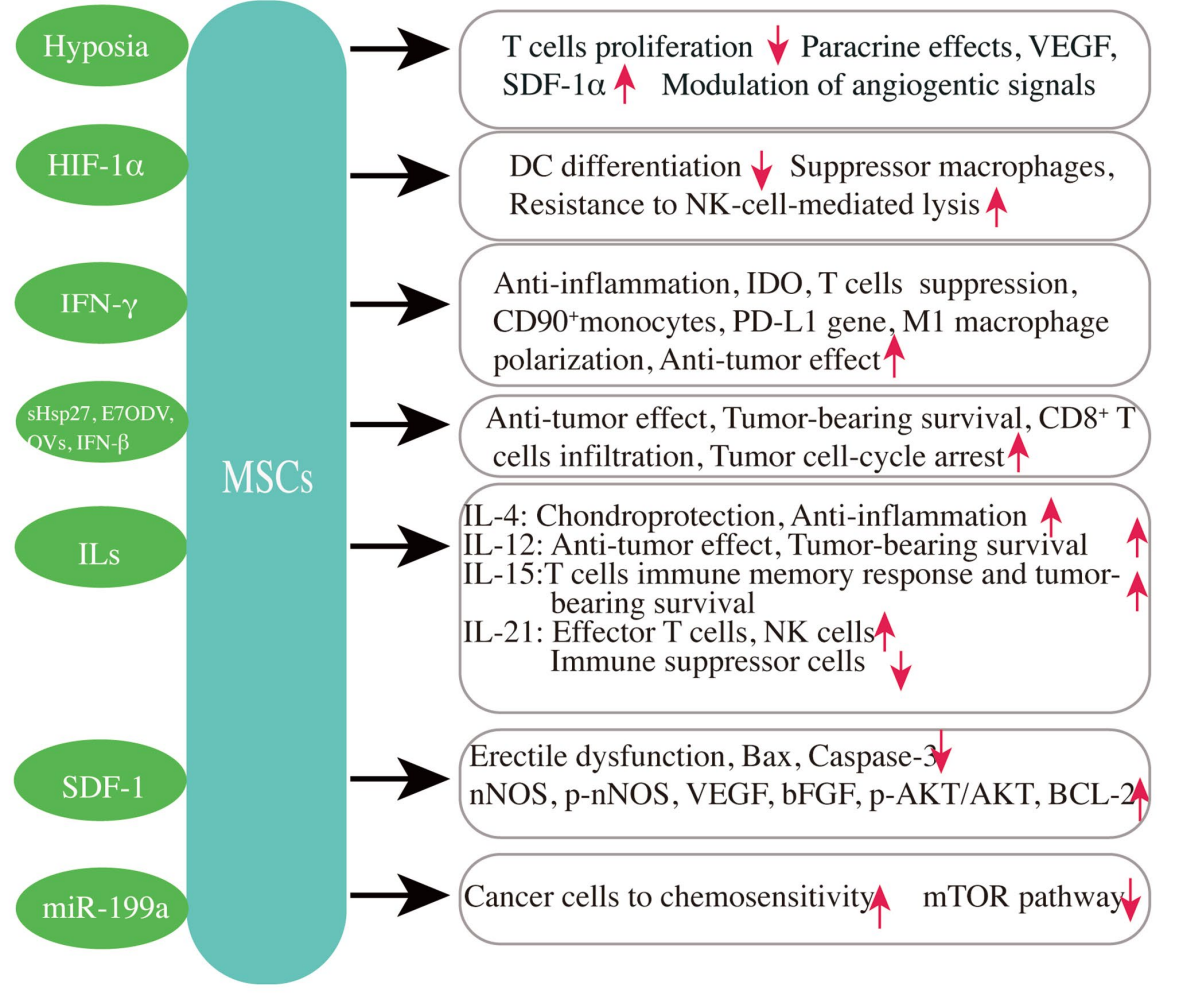
**Modified MSCs to increase their immunomodulatory functions and therapeutic efficacy**. MSCs exert better immunomodulatory effects through modification compared with signal MSCs admin-istration. MSCs pretreated with hypoxia. heat shock. inflammatory stimulation, and et al. have the poten-tial to increase their therapeutic potency, the expression of cytoprotective genes, and secretion of repara-tive factor. Abbreviations: IL, intcricukin; IFN, interferon; IDO, Indolcaminc 23-dioxygcnasc; Bax, 13cl-2-associated x; nNOS, neuronal nitric oxide synthase; VEGF, vascular endothelial growth factor; bFGF, basic fibroblast growth factor; BCL-2, B-cell lymphoma-2; AKT, protein kinase B; HIF-1α, hypoxia-inducible factor 1α; sHsp27, heat shock proteins 27; E7ODV, E7 oncoprotein DNA vaccinations; OVs, oncolytic viruses; SDF-1, stromal cell-derived factor-1

Additionally, MSCs as a possible means can deliver oncolytic viruses (OVs), which can overcome host antiviral immunity [159]. MSCs armed by oncolytic herpes simplex virus (oHSV) successfully delivered viral progeny to target melanoma brain metastasis, ultimately inhibiting tumor growth rate and prolonging survival of brain tumor-bearing mice [159, 160]. Although many research examples of immunomodulation and delivery are successful, we still need more work. The promising strategies that are commonly being addressed are displayed in Fig. 3.

## MSCs-induced Immunomodulation in Clinical Studies and Trials

39 studies have been performed to investigate the immunomodulation of MSCs in clinical trials. The diseases they focus on are infections, diabetes, knee osteoarthritis, relapsing-remitting multiple sclerosis (RRMS), Crohn’s Disease and etc. which represented the most common immunes system diseases in the clinical (Table 2). A recent phase I/IIa trial (Identifier: https://clinicaltrials.gov/ct2/show/NCT02351011) utilized a 50 million dose of BMSCs per person to 12 patients with late-stage Kellgren‐Lawrence knee osteoarthritis. The results suggested that the administration of autologous BMSCs derived from BM of the posterior superior iliac spine is effective in quality of life, safe for intra‐articular injection with no serious adverse events, and contribute to the improvements of synovial inflammation and pain [161]. Likewise, a conducted phase II/III study (Clinical Trial Number: (UMIN-CTR Numberiii: UMIN000006719) indicated that BMSC transplantation enhanced the overall survival rate in twenty-five patients with acute GVHD (steroid-refractory grade III or IV) with no observed adverse events [162]. In an open-label phase IIa study (Clinical Trial Number: NCT00395200), autologous BMSCs with a dose of 1.6 × 10^6^ cells per kg bodyweight were safely administrated to patients with secondary progressive multiple sclerosis (SPMS) and the improvement of structure, function, and physiology demonstrated the neuroprotective effects [163]. In phase I/IIa studies for 34 adult patients with atopic dermatitis from moderate to severe (AD), subcutaneously infused high-dose HUC-MSCs (5.0 × 10^7^cells) therapeutically improved the disease features, suggesting the significant reduction of severity scoring and eczema area, severity index for atopic dermatitis, pruritus score, serum IgE levels and the number of blood eosinophils [164]. A pilot study associated with serial intrafistular administration of autologous BMSCs for 12 consecutive outpatients with refractory fistulizing Crohn’s disease demonstrated that local BMSCs therapy is feasible, safe, and beneficial for this disease by modulating mucosal T cell apoptotic rate and 7 patients with sustained complete closure of fistula tracks were observed in 12 patients [165]. A triumphant randomized and double-blind controlled phase III clinical trial (ClinicalTrials.gov, registered number NCT01541579) utilizing expanded allogeneic AMSCs (a single intralesional injection of 1.2×10^9^ cells) to treat 212 Crohn’s disease patients possessing refractory complex perianal fistulas suggested that a restorative period of at least 24 h post-thawing culture promoted MSCs’ quality [166–169].

**Table 2:** The immunomodulatory behaviour of MSCs in recent clinical and preclinical studies

Disease	Origin of MSCs	Treatment routes	Identified Immunomodulatory Effect		References	
Preclinical research						
Ovarian cancer mice model	Mice compact bone MSCs	Intraperitoneal injection	Activation of CD4^+^ and CD8^+^ T cells and the inhibition of Tregs		[88, 89]	
kidney allograft	Mice BMSCs	Intravenous injection	Suppression of T cells and improvement of Tregs generation		[114]	
Septic Mice	HUC-MSCs	Intravenous injection	Improving anti-inflammatory effects, increasing the Treg cells, promoting macrophage phagocytosis, enhancing the reparative macrophage, secreting more VEGF		[171]	
Asthmatic mice	Mice BMSCs	Intraperitoneal injection	Better immunomodulatory effect		[172]	
Mice With acute liver failure	Mice AMSCs	intravenous injection	Removeing debris and improving IL-6 liver regeneration		[173]	
Mice inflammatory bowel disease (IBD)	Intestine Peyer’s patch MSCs	Intravenous injection	Improve the symptoms of mice IBD model		[174]	
GVHD mice	Human MSCs	intravenous injection	Engagement of PD-1 ligands, reduction of the clinical score and prolonging the survival of mice		[175]	
Acute respiratory distress syndrome (ARDS) mice	HUC-MSCs	Intravenous injection	Ameliorated lung injury of ARDS and inactived YAP to facilitate type II alveolar epithelial cells (AECII) differentiation.		[176]	
Mice alkali-burn injury model	Mice BMSCs	Local injection	Enhancement of their immunomodulatory and pro-angiogenic capacity, decrease of pro-inflammatory IL-1β		[177, 178]	
Host’s immune system	HUC-MSCs	Intravenous injection	Elevation of neutrophil percentage and concentration of neutrophil chemoattractants		[179]	
**Clinical Trials**						
**Disease**	**Origin of MSCs**	**Treatment routes**	**Clinical Trial Number**	Phase	Identified Immunomodulatory Effect	**References**
Osteoarthritis	Autologous BMSCs	Intra-articular infusion	NCT02351011	I/II clinical trial	Decrease of synovial inflammation and pain	[161]
Steroid-refractory grade III or IV acute GVHD	Autologous BMSCs	Intravenous injection	UMIN000006719	II/III clinical trial	Enhancement of the overall survival rate	[162]
Multiple sclerosis	Autologous BMSCs	Intravenous injection	NCT00395200	IIa clinical trial	Improvement of structure, function, and physiology	[163]
Moderate to severe atopic dermatitis	HUC-MSCs	Subcutaneous injection	NCT01927705	I/II clinical trial	Reduction of severity scoring and eczema area, severity index for atopic dermatitis, pruritus score, serum IgE levels and the number of blood eosinophils	[164]
Crohn’s disease	Autologous BMSCs	Intrafistular injections	/	/	A feasible, safe and beneficial therapy	[165]
Crohn’s disease	Allogeneic AMSCs	Intrafistular injections	NCT01541579	III clinical trial	Long-term Efficacy and Safety	[166–169]
Gastrointestinal adenocarcinoma	Autologous BMSCs	Intravenous injection	European Union Clinical Trials Register 2012-003741-15	I/II clinical trial	Favorable safety and tolerability	[180]

However, it is evident that the induction of some side effects may be mediated by MSCs-related treatments. For instance, recent preclinical research demonstrated that the acute hypothermia of the brain microenvironment moderated the function of intranasal administered MSCs, enhancing long-lasting motor-cognitive deficits and endothelial activation, resulting in a pro-inflammatory environment, increasing the peripheral immune cell infiltration, which exacerbated the brain injury in the hypoxic-ischemic brain [170].

## Hopeful Strategies have the Potential to Improve the MSCs Efficacy of Future Trials

Preclinical reports have suggested that different administrated ways and modified factors of MSCs have the therapeutical potential for different diseases. Recent studies have demonstrated that MSCs homing to injury sites by intravenously delivering MSCs or depending on migration and mobilization of endogenous MSCs was investigated in a feeder layer-based transwell-based model. The overexpression of fibroblast growth factor 21 (FGF21) in MSCs (FGF21-MSC) exhibited the improvement of the MSCs’ homing ability to injury sites in a traumatic brain injury (TBI) mouse model [181]. Analogously, colony-stimulating factor 2 (CSF-2), a hematopoietic growth factor, improved the differentiation and migratory capacity of MSCs and promoted the therapeutic effects via PI3K/AKT and/or FAK/ERK1/2 signal axis [182]. A proinflammation cytokine IL-1β-mediated more production of matrix metalloproteinase-1 (MMP-1) improved the migration of MSCs through the activation of the protease-activated receptor 1 (PAR1) and G-protein-coupled signaling pathways [183]. Intriguingly, intranasally administered exosomes derived from MSCs particularly accumulate as an inflammatory-driven style in injured brain sites of relevant murine models for up to 4 days post-administration, which is highly associated with the neuro-inflammatory signals in the various brain pathological regions of different diseases, such as stroke, autism, Parkinson’s disease, and Alzheimer’s disease [184]. Hypoxic preconditioning as a boosting factor enhanced MSCs’ migration ability and survival. Preconditioned MSCs with hypoxia evoked an increased LincRNA-p21 production, pivotal in MSCs homing through the HIF-1α/CXCR4 and CXCR7 pathway in vitro [185].

Another strategy to improve the MSCs’ effectiveness is to facilitate the ways of their cryopreservation. More specifically, freshly thawed MSCs following cryopreservation stunted functional characteristics, including the upregulation of metabolic activity and apoptosis and downregulation of cell proliferation, immunosuppressive capabilities, clonogenic capacity, and critical regenerative genes [14, 186, 187]. Notably, a 24-hour acclimation period reactivated the thawed cells, diminishing apoptosis and enhancing the high-mobility group box (HMOX-1) and tumor necrosis factor-stimulated gene-6 (TSG-6) genes expression with anti-inflammatory effects and VEGF gene expression with angiogenic effects [186]. Consequently, MSCs should be standardized to implement robust quality and reliability, particularly in large-scale frozen MSCs products. Recent research results concluded that administering two freezing steps accompanied by a preceding cell-culture phase of at least one passage did not impair the essential quality or parameter attributes of the MSCs’ ultimate cryopreserved and thawed product [187]. A triumphant randomized and double-blind controlled phase III clinical trial (ClinicalTrials.gov, registered number NCT01541579) utilizing expanded allogeneic AMSCs (a single intralesional injection of 1.2×10^9^ cells) to treat 212 Crohn’s disease patients possessing refractory complex perianal fistulas suggested that a restorative period of at least 24 h post-thawing culture promoted MSCs’ quality [166–169]. Hence, a 24-hour acclimation period of culture for cryopreserved cells is crucial to reactivate the thawed MSCs for restoration of their impaired MSCs’ capabilities.

## Conclusions and Future Perspectives

Due to MSCs’ tropism with other cell types and immunomodulatory attributes, the administration of MSCs has been a novel therapeutic strategy in preclinical and clinical research. Due to the immunomodulatory capability of MSCs modified by the mutual effect and various inflammatory cytokines with other immune cells, MSCs could mediate the recovery of various inflammatory disorders. The major mechanisms associated with the immunomodulatory capability of MSCs include intercellular liaison and paracrine activity, in which process MSCs are modified by inflammatory stimuli, extracellular vesicles, cytokines, chemokines, or co-culture with other immune cells. Consequently, the transplantation of MSCs can be made an administrable, potential, and feasible method of cell-free therapy. However, it is still hard to understand the mechanisms of how the variability of MSC impacts their immunomodulatory capability. The administration of MSCs likely mediates highly intricate immunomodulatory mechanisms through their secretome. The following work should explore the effects of chronic inflammatory cytokines and other factors on MSCs-induced immunomodulatory effects. These methods can identify new preconditioned approaches in promoting the therapeutic efficacy of MSCs and weaken the variation of their paracrine capability, especially in clinical application.

As for utilizing MSCs as a promising strategy in clinic translation, many investigators have mainly focused on gene delivery vehicles to improve the immunomodulation of MSCs. As modified MSCs delivering targeted molecules and genes have been utilized in multiple immune diseases of clinical trials, it is critical to identify the targets to develop available MSCs therapies in the diseased microenvironment/cells. For instance, previous reports have demonstrated that MSCs modified with a bi-functional molecule consisting of epidermal growth factor receptor-targeted nanobody and death receptor-targeted ligand TRAIL (ENb-TRAIL) significantly increase survival and alleviate tumor burden through the mechanisms of targeting receptor-induced proliferation and death pathways in multiple tumor types [188]. Similarly, UCMSC engineered with Tandab (CD3/CD19) combined with IDO pathway inhibitor D-1-methyl-tryptophan (D-1MT) can be utilized as an efficient therapeutic approach to inhibit tumor growth significantly in vivo by treating lymphoma of B cells [189].

A real challenge faced by MSCs research is the conflict between in vitro and in vivo results. Liu et al. reported that osteogenic cells (OCs) differentiated from MSCs possessed their immunomodulatory ability and were immune-privileged in vitro. However, such immunomodulatory and immune-privileged abilities were lost after OCs implantation in vivo experiments following an osteogenesis model of New Zealand white rabbit [190]. A study from Isakova et al. found that the engraftment of allogeneic MSCs had weakly immunogenic after post-transplantation compared with autologous MSCs in a rhesus macaque model, which limited their durable engraftment levels [191]. These discrepancies indicate the significance of observing the MSCs’ immunogenicity after transferring from in vitro to in vivo experiments and it is critical to maintaining an effective therapy in further clinical applications. Eventually, by enhancing immunomodulatory potential, further investigating homing mechanisms, and improving cryopreservation techniques accompanied by detailed preclinical and clinical studies, MSCs with promising potential can be utilized in future clinical settings.

## Data Availability

Not applicable.
